# Building Efficient CNN Architectures for Histopathology Images Analysis: A Case-Study in Tumor-Infiltrating Lymphocytes Classification

**DOI:** 10.3389/fmed.2022.894430

**Published:** 2022-05-31

**Authors:** André L. S. Meirelles, Tahsin Kurc, Jun Kong, Renato Ferreira, Joel H. Saltz, George Teodoro

**Affiliations:** ^1^Department of Computer Science, Universidade de Brasília, Brasília, Brazil; ^2^Biomedical Informatics Department, Stony Brook University, Stony Brook, NY, United States; ^3^Department of Mathematics and Statistics and Computer Science, Georgia State University, Atlanta, GA, United States; ^4^Department of Computer Science, Universidade Federal de Minas Gerais, Belo Horizonte, Brazil

**Keywords:** digital pathology, deep learning, CNN simplification, tumor-infiltrating lymphocytes, efficient CNNs

## Abstract

**Background:**

Deep learning methods have demonstrated remarkable performance in pathology image analysis, but they are computationally very demanding. The aim of our study is to reduce their computational cost to enable their use with large tissue image datasets.

**Methods:**

We propose a method called Network Auto-Reduction (NAR) that simplifies a Convolutional Neural Network (CNN) by reducing the network to minimize the computational cost of doing a prediction. NAR performs a compound scaling in which the width, depth, and resolution dimensions of the network are reduced together to maintain a balance among them in the resulting simplified network. We compare our method with a state-of-the-art solution called ResRep. The evaluation is carried out with popular CNN architectures and a real-world application that identifies distributions of tumor-infiltrating lymphocytes in tissue images.

**Results:**

The experimental results show that both ResRep and NAR are able to generate simplified, more efficient versions of ResNet50 V2. The simplified versions by ResRep and NAR require 1.32× and 3.26× fewer floating-point operations (FLOPs), respectively, than the original network without a loss in classification power as measured by the Area under the Curve (AUC) metric. When applied to a deeper and more computationally expensive network, Inception V4, NAR is able to generate a version that requires 4× lower than the original version with the same AUC performance.

**Conclusions:**

NAR is able to achieve substantial reductions in the execution cost of two popular CNN architectures, while resulting in small or no loss in model accuracy. Such cost savings can significantly improve the use of deep learning methods in digital pathology. They can enable studies with larger tissue image datasets and facilitate the use of less expensive and more accessible graphics processing units (GPUs), thus reducing the computing costs of a study.

## 1. Introduction

Pathology image analysis is quickly evolving thanks to advances in scanner technologies that now enable rapidly digitizing glass slides into high resolution whole slide images (WSIs). This has also been followed by several developments in computer aided diagnosis analysis tools and methods, which have improved the use of information computed from tissue characteristics in WSIs in disease classification, prediction of clinical outcomes, etc. (1–3). Deep learning methods have demonstrated significant improvements over traditional machine learning and other image analysis methods in a wide range of tissue image analysis tasks (4–10). Consequently, deep learning-based image analysis is rapidly becoming a mainstream approach in digital pathology.

The advances attained with the deep learning methods have also been accompanied by multiple challenges in order to make them more routinely used in pathology image analysis. For instance, these methods require a significant amount of annotated data to be used in training, which is particularly costly in digital pathology as it requires an expert pathologist to manually annotate large volumes of data (11, 12). Also, applications developed with deep learning should consider explainability to improve confidence in their use (13, 14).

We address another challenge with application of deep learning in digital pathology; the high computational cost of deep learning inference, which has adversely impacted the effective use of deep learning in many application domains (15). This problem is particularly more pronounced in digital pathology because WSIs are extremely high resolution images (in the range of 100K× 100K pixels). A study analyzing thousands of WSIs would require substantial computing capacity. High computing requirements can significantly limit the use of deep learning in research and as a routine component of digital pathology workflows.

The demanding computational costs of deep learning models can be addressed by CNN simplification and acceleration techniques, such as: network pruning (16–18), sparsification (19, 20), quantization (21, 22), etc. Among network pruning solutions, there are those that concentrate on removing filters in the convolutional layers, which are referred to as channel or filter pruning (23–25). Other techniques act on a broader range of structures, removing full layers or even blocks of layers (26).

Network pruning solutions have been the focus of a number of publications, presenting good results in CNN speedup and also enabling lossless model compression (27). Filter pruning techniques and network pruning in general offer varying possibilities to select which filters from which layers should be excluded from the network or which structures to be removed. However, this is not performed in a balanced manner taking into consideration all model dimensions together, which may limit the performance and accuracy of the reduced network (28–30).

In this work, we present a novel approach that can generate more efficient Convolutional Neural Network (CNN) architectures to speed up the execution of model training and inference. Our approach, called Network Auto-Reduction (NAR), performs transformations in a given CNN architecture in order to reduce its width, depth, and resolution dimensions (also called components) to generate a novel architecture with the desired computational cost (in terms of number of FLOPs) and with minimal loss of accuracy. This simplification employs a compound scaling method with a set of fixed scaling coefficients. The goal is to maintain a balance among the components of the network—for instance, a larger input resolution would require more receptive fields and a larger number of channels to capture details of the input image as is theoretically shown in (28). NAR differs from most of the previous works that focus on reducing a single or a couple of the dimensions of the network (25, 27, 29, 31–33).

We experimentally evaluate our approach in a real-world application that classifies tumor-infiltrating lymphocytes (TILs) in WSIs (34, 35) (presented in Section 2.1). TILs are a type of white blood cells in the immune system, whose patterns found in the tissue images have been shown to have consistent correlations with patient overall survival in multiple cancer types (36–40). In our evaluation, we use ResNet50 V2 and Inception V4 as full, baseline networks and simplify them with NAR. We compare NAR to a state-of-the-art method, called ResRep (27). ResRep is designed to carry out lossless channel pruning (filter pruning) to slim down a CNN through a reduction in the width or number of output channels of convolutional layers. The experimental evaluation shows that NAR can generate CNNs with demands up to 4 × lower than the original CNN, while delivering the same classification quality (AUC). The simplified networks generated by NAR are more efficient, with smaller requirements for the same AUC values when compared with the networks generated by ResRep.

The rest of this document is organized as follows: Section 2 presents the motivating TIL classification application, the NAR strategy proposed here and summarizes the ResRep approach. Section 3 shows the performance evaluation in detail and Section 4 discusses the main finds and promising directions for future work.

## 2. Materials and Methods

### 2.1. Tumor-Infiltrating Lymphocytes (TIL) Classification Using Deep Learning

This work is motivated by analyses carried with deep learning models of WSIs to identify and classify spatial patterns of TILs (34, 35). There is increasing evidence that TIL patterns in cancer tissue correlate with clinical outcomes; for example, high densities of TILs indicate favorable outcomes, such as longer survivals for patients (37). Quantitative analyses of TIL patterns can provide valuable information about interactions between cancer and immune system and novel bio-markers for prediction of prognosis and treatment response.

WSIs allow a researcher to carry out quantitative investigations of the tumor microenvironment at the subcellular level. This has motivated the development of image analysis methods to extract and characterize quantitative imaging features from WSIs (1–3, 41, 42). Deep learning methods based on Convolutional Neural Networks (CNNs) have emerged as an effective approach for image analysis in several domains. CNNs have been employed for a variety of tissue image analysis tasks, including object identification, segmentation, and recognition of spatial patterns (34, 43–49).

**Figure 1:**
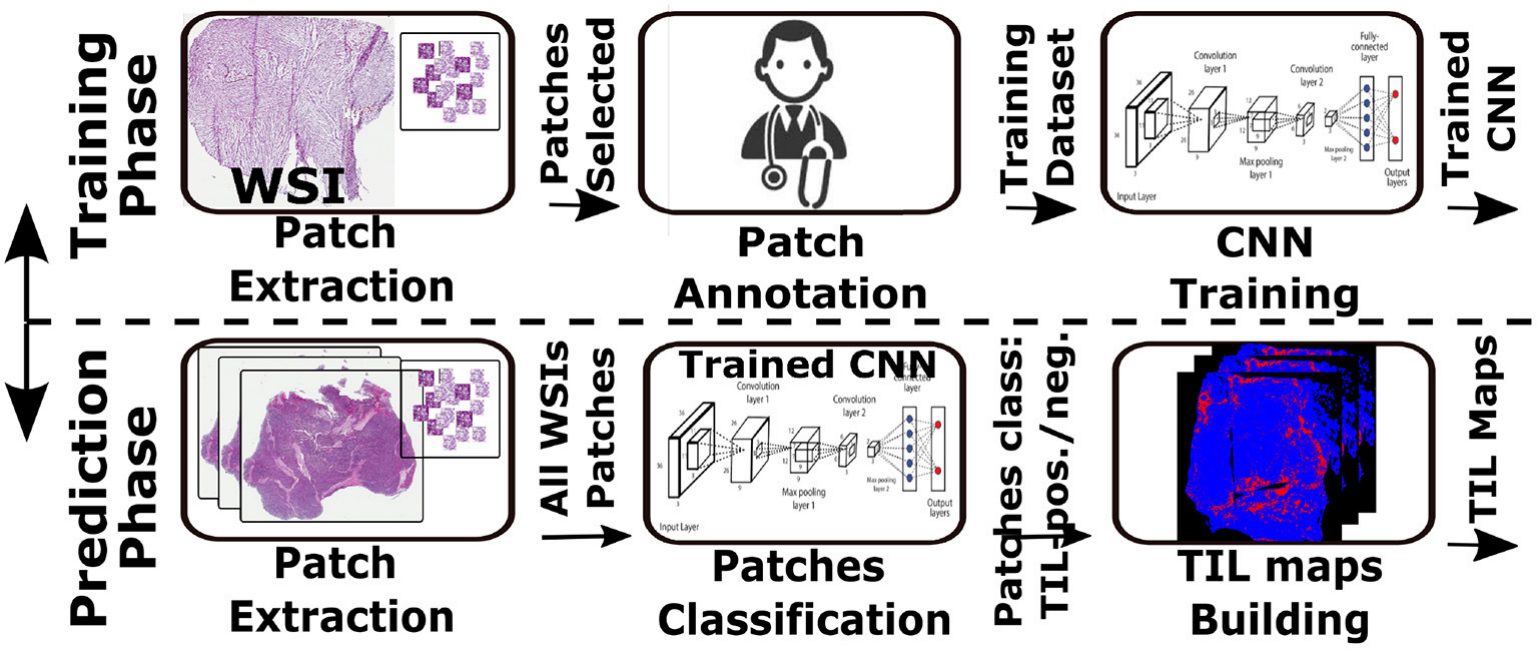
Use-case TIL analysis workflow. CNN is trained to identify TIL rich tissue based on patches annotated by expert pathologist (top). The CNN model is then used to classify input WSI in a patch basis. The result is a TIL map presenting TIL rich regions in the input tissue.

Figure 1 shows a TIL analysis pipeline, based on the work done in (34), that predicts distributions of TILs in images of hematoxylin and eosin (H&E) stained tissue specimens. In this pipeline, an input image is partitioned into small patches—the size of a patch is 50 × 50 square microns in our application. A CNN classification model classifies the patches into TIL-positive and TIL-negative classes (a binary classification operation). As is shown in the figure, the pipeline is composed of a training phase and a prediction phase. In the training phase (shown in the top), the CNN learns to classify input image patches. In this process, patches are extracted from multiple WSIs, pathologists review and annotate them, and the CNN classification model is trained. The selection of patches and model training is repeated until the desired accuracy level is reached. The prediction phase (bottom part of the image) applies the trained model to input patches from unseen WSIs to compute TIL maps that identify tissue regions with TILs—TIL-positive patches are shown as Red dots on a Blue background, which represents tissue.

While CNNs have been applied successfully for TIL analysis (34, 35), scaling the analysis to thousands of WSIs is challenging, because of the CNNs high computational cost. This poses a major limitation to a broader adoption of CNN-based methods in the digital pathology domain. We propose a method that intelligently simplifies a CNN to reduce its computational cost while minimizing loss of model accuracy. The proposed method is discussed in the next section.

### 2.2. Network Auto-Reduction (NAR)

We propose Network Auto-Reduction (NAR) to simplify CNNs and reduce their execution cost in the inference (prediction) phase. Several approaches have been proposed for CNN simplification. Most of the prior approaches aim to reduce one of the dimensions of the CNN: depth, width or input resolution (27). Some studies proposed removing specific CNN filters (25, 29, 31–33), or introducing weight sparsity (18) or applying a combination of both (26, 27). In most of those cases, the CNN is re-trained multiple times while the reduction operations are iteratively applied. This is computationally expensive and may not even be feasible in applications that employ large training datasets.

**Figure 2:**
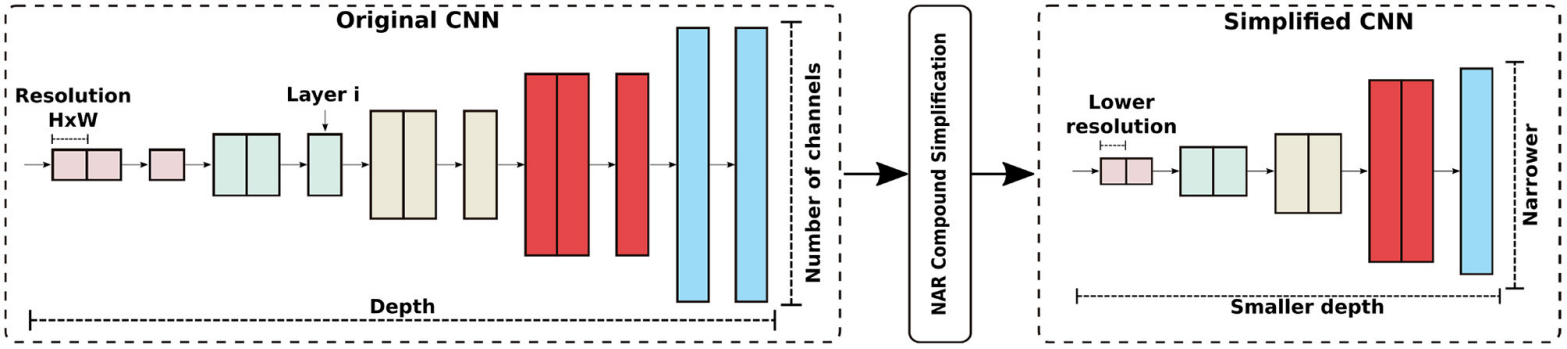
NAR compound CNN simplification modifies depth, width, and input resolution in order to have a balance among CNN components.

NAR simplifies a CNN by modifying the depth, width, and input resolution of the model together. The goal is to maintain a balance between network building blocks in order for the simplified CNN to attain good accuracy, as demonstrated in previous work (28, 50, 51). The compound simplification process is illustrated in Figure 2.

Our method is inspired by the approach proposed by Tan et al. (52) to scale up simple CNNs. Their method was designed to increase the size of a simple CNN in order to improve its prediction performance. Here, on the other hand, we address the problem of simplifying a CNN that is already known to perform well in the target domain, but has a high computation cost. Tan et al. (52) formulated the problem of scaling-up a CNN as an optimization problem defined in Equation (1), given a target memory consumption (TM) and (TF):


(1) $$\begin{array}{l} \;max_{d,w,r}\;Accuracy(M(d,w,r))\\ \;s.t\;M(d,w,r)\;=\;\underset{i=1,...,s}{⊙}{\hat{ℱ}}_{i}^{d.{\hat{L}}_{i}}\left(X_{\langle r.{\hat{H}}^{i},r.{\hat{W}}^{i},w.{\hat{C}}^{i}\rangle }\right)\\ Memory(M)\;\leq \;TM;\\ \;(M)\;\leq \;TF, \end{array}$$

Here, ⊙_*i* = 1, ..., *s*_ is the composition of the layers of a given CNN *M*. Each layer *i* can be viewed as the application of function ${\hat{F}}_{i}$ on its input tensor *X*_*i*_, with dimensions Ĥ^*i*^, Ŵ^*i*^, Ĉ^*i*^ (height, width, channels). The layers can be repeated in a sequence of ${\hat{L}}_{i}$ occurrences. The transformation process changes all three components of a network simultaneously, *depth* (the number of layers ${\hat{L}}_{i}$), *width* (the number of channels Ĉ_*i*_) and *resolution* (the height Ĥ_*i*_ and width Ŵ_*i*_ of tensor *X*_*i*_) in a balanced way. The scaling coefficients *d, w, r* used by Tan et al., enabled creating a bigger network *M* with $d.{\hat{L}}^{i}$ occurrences of layer *i* and input size *r*.Ĥ^*i*^, *r*.Ŵ^*i*^, *w*.Ĉ^*i*^, except for layer *i* = 0, in which the input dimensions are the same as the input image dimensions and channels. For given values of *d, w, r*, the cost of the scaled-up CNN is increased proportionally to *d*.*w*^2^.*r*^2^.

According to Tan et al., it is critical to balance the scaling coefficients in order to obtain the best accuracy/efficiency relation for a given resource constraint. To that end, a uniform compound scaling strategy is used to distribute the cost increase among these parameters through a ϕ coefficient, such that *d* = α^ϕ^, *w* = β^ϕ^, and *r* = γ^ϕ^ with a restriction that α.β^2^.γ^2^ ≈2. The values of α, β, and γ that produce the best accuracy are determined by a model grid search (52).

In NAR, we apply a reduction factor to each CNN component such that *d* = α^−ϕ^, *w* = β^−ϕ^, *and r* = γ^−ϕ^ with the same restriction valid for α, β, and γ. This results in a theoretical reduction of $\frac{1}{2^{\phi }}$ for every value of ϕ. Therefore, NAR generates reduced versions of any block based CNN.

### 2.3. ResRep

ResRep (27) is a state-of-the-art CNN pruning strategy that uses structural re-parameterization to reduce a network's width. It implements a two step solution, referred to as *remembering* and *forgetting* steps inspired by neurobiology research. In the remembering step, the network is trained with the addition of *compactor* layers attached to the original convolutional layers. The goal is to identify filters that contribute little to the learning process. The compactors are 1 × 1 convolutional layers that apply gradient penalties, making some channels' gradients approach zero. The forgetting step is executed after the remembering step and reconstructs the original model based on the compactor trained network, but without some channels.

A key feature of ResRep is the mechanism by which channels are selected to be removed from the original network. The selection process uses a “gradient resetting” scheme, applied to the compactors' gradients only. A group Lasso penalty is used in conjunction with the training objective function to produce a channel-wide sparsity. The gradient resetting operation is formulated in Equation (1).


(2) $$\begin{array}{r} L_{total}\left(X,Y,\text{Θ}\right)\;=\;L_{perf}\left(X,Y,\text{Θ}\right)\;+\text{ λ}P\left(K\right) \end{array}$$

(3) $$\begin{array}{l} G(F)\;=\frac{\;\partial L_{total}(X,Y,\text{Θ})}{\partial F}\leftarrow \frac{\partial L_{perf}(X,Y,\text{Θ})}{\partial F}\\ {}*m\;+\text{ λ}\frac{F}{||F|{|}_{E}}. \end{array}$$

Here, *L*_*total*_ is the objective function applied to input X with label Y, given current network weights Θ. The λ is a penalty strength factor and *P*(*K*) is the Lasso penalty added to the regular cost function *L*_*perf*_. The gradients for each filter (**F**) of the convolutional layer may be zeroed with a binary mask *m*. The final gradient *G*(**F**) is compared to a threshold value (ϵ). If it is below the threshold, the filter is removed. It is expected that *G*(**F**) will be close to zero for filters for which the binary mask *m* is 0, since only the penalties are considered.

## 3. Results

The network cost reduction techniques were evaluated with the TIL classification application described in Section 2.1 and two popular CNN architectures, ResNet50 V2 (53) and Inception V4 (54)—the two CNNs had been successfully employed for whole slide image analysis in a previous work (35). The CNNs were trained with 4,300 image patches extracted from a set of 56 WSIs from 10 tumor tissue types, including breast, prostate and pancreatic cancer, in The Cancer Genome Atlas (TCGA) repository (55). Fifteen thousand patches extracted from another set of 5 WSIs comprised the test dataset. The full list of the WSIs is given in Supplementary Tables 3, 4, which also includes the percentage of TIL positive patches in each WSI. The images were downloaded in their native Aperio SVS file format. SVS files have a hierarchical representation that stores multiple resolutions of the same image. We used the highest resolution available for each WSI. If an image is obtained at 40x or 20x magnifications, the physical dimensions of a pixel are 0.25×0.25 μm or 0.5×0.5 μm, respectively. We employed the OpenSlide library (http://openslide.org/formats/aperio/) to read the images and extract patches. The images along with their TIL classification (Map) are publicly available (https://cancerimagingarchive.net/datascope/TCGA_TilMap/).

In all of the original and simplified CNN configurations, an input image patch covers a tissue area of 50×50μm, which was resized to the expected input image size of each CNN. The number of patches that a CNN has to process to analyze a WSI is the same as the other CNNs, regardless of the input size required by each CNN.

The deep learning models were trained and tested on a machine running Linux, equipped with 2 Intel Xeon Gold 6248 “Cascade Lake” CPUs (with 20 cores each), 512 GB of DDR4 RAM, and an NVIDIA Tesla V100 GPU with 32 GB of dedicated memory. In all of the experiments, the models were trained from scratch for a varying number of epochs (50 for NAR and 180 for ResRep, which requires a larger number of epochs to simplify the CNN) using Adam optimization algorithm, a learning rate of 0.0005, and weight decay of 0.0005. StepLR was used as learning rate scheduler for ResRep, with step size of 5 epochs and gamma as 0.5 (learning rate reduction factor). In addition to NAR and ResRep, we have also evaluated a reduction strategy in which only the input image is reduced. This strategy is called input reduction (IR). With IR, we evaluated the impact of the compound reduction implemented by NAR against input data reduction only. The IR strategy results in smaller feature maps in memory but does not require changes to the CNN architecture, which remains exactly the same as the original.

The classification performances of the models trained with the simplified CNNs generated by ResRep and NAR were evaluated using the Area Under the ROC Curve (AUC) metric, the values of which were computed as the mean of values from 3 runs. The values of α = 1.2, β = 1.1 and γ = 1.15 used here that lead to the best performance were determined using a grid search (52). The execution cost of each model was measured in terms of the number of Giga- (G) required to process a given input patch covering an area of 50×50μm. The total count considers both convolutional and dense layers, given, respectively, by the relations *F*_*conv*_ = 2*Number of channels*Kernel shape*Output shape and *F*_*dense*_ = 2*Input size*Output size. The NAR codes were developed using Keras and Tensorflow, while ResRep was implemented with PyTorch.

### 3.1. Simplification of ResNet50 V2 by NAR, ResRep, and IR

This set of experiments compare NAR, ResRep, and the Input Reduction (IR) approaches in simplifying the ResNet50 V2. The value of ϕ in NAR was varied between 1 and 3. Values greater than 3 generated simplified architectures that were purely sequential models that did not resemble the original model at all. Moreover, ϕ = 3 resulted in significant drop in classification performance.

The simplified CNNs generated by different configurations of NAR and ResRep are summarized in Tables 1, 2, respectively. As is shown in Table 1, NAR reduces multiple components of the network; this is illustrated by different number of blocks in each stage and different filter quantity in each convolutional layer. ResRep, on the other hand, primarily prunes the filters in the last stages of the network. In Table 2, *P* marks positions where filters have been pruned. The filters in stage 2 are not pruned until ϵ = 0.90 and no filters are pruned in stage 1.

**Table 1:** Number of parameters and layers organization in original ResNet50 V2 and NAR simplified networks.

**CNN**	**ORIGINAL**	**NAR **ϕ = 1****	**NAR **ϕ = 2****	**NAR **ϕ = 3****
ResNet50 V2 (53)				
# Params	23,568,898	14,583,140	11,274,413	8,514,988
Conv 1	7 ×7, 64, stride 2			
Stage 1	$\left[\begin{array}{c} 1\times 1,64\\ 3\times 3,64\\ 1\times 1,256 \end{array}\right]$ × 3	$\left[\begin{array}{c} 1\times 1,58\\ 3\times 3,58\\ 1\times 1,232 \end{array}\right]$ × 2	$\left[\begin{array}{c} 1\times 1,53\\ 3\times 3,53\\ 1\times 1,212 \end{array}\right]$ × 2	$\left[\begin{array}{c} 1\times 1,48\\ 3\times 3,48\\ 1\times 1,192 \end{array}\right]$ × 2
Stage 2	$\left[\begin{array}{c} 1\times 1,128\\ 3\times 3,128\\ 1\times 1,512 \end{array}\right]$ × 4	$\left[\begin{array}{c} 1\times 1,106\\ 3\times 3,106\\ 1\times 1,424 \end{array}\right]$ × 3	$\left[\begin{array}{c} 1\times 1,116\\ 3\times 3,116\\ 1\times 1,464 \end{array}\right]$ × 3	$\left[\begin{array}{c} 1\times 1,96\\ 3\times 3,96\\ 1\times 1,384 \end{array}\right]$ × 2
Stage 3	$\left[\begin{array}{c} 1\times 1,256\\ 3\times 3,256\\ 1\times 1,1024 \end{array}\right]$ × 6	$\left[\begin{array}{c} 1\times 1,233\\ 3\times 3,233\\ 1\times 1,932 \end{array}\right]$ × 5	$\left[\begin{array}{c} 1\times 1,212\\ 3\times 3,212\\ 1\times 1,848 \end{array}\right]$ × 4	$\left[\begin{array}{c} 1\times 1,192\\ 3\times 3,192\\ 1\times 1,768 \end{array}\right]$ × 3
Stage 4	$\left[\begin{array}{c} 1\times 1,512\\ 3\times 3,512\\ 1\times 1,2048 \end{array}\right]$ × 3	$\left[\begin{array}{c} 1\times 1,465\\ 3\times 3,465\\ 1\times 1,1860 \end{array}\right]$ × 2	$\left[\begin{array}{c} 1\times 1,423\\ 3\times 3,423\\ 1\times 1,1692 \end{array}\right]$ × 2	$\left[\begin{array}{c} 1\times 1,385\\ 3\times 3,385\\ 1\times 1,1540 \end{array}\right]$ × 2

*The parameter count considers a binary classification problem*.

**Table 2:** Number of parameters and layers for the ResRep reduced networks (binary classification).

**CNN**	**ϵ = 0.82**	**ϵ = 0.84**	**ϵ = 0.86**	**ϵ = 0.88**	**ϵ = 0.90**	**ϵ = 0.92**	**ϵ = 0.94**
ResNet50 V2 (53)							
# Params	12,527,836	9,421,008	8,663,740	9,225,475	7,931,287	4,882,052	4,696,612
Conv 1	7 × 7, 64, stride 2						
Stage 1	$\left[\begin{array}{c} 1\times 1,64\\ 3\times 3,64\\ 1\times 1,256 \end{array}\right]$ × 3	$\left[\begin{array}{c} 1\times 1,64\\ 3\times 3,64\\ 1\times 1,256 \end{array}\right]$ × 3	$\left[\begin{array}{c} 1\times 1,64\\ 3\times 3,64\\ 1\times 1,256 \end{array}\right]$ × 3	$\left[\begin{array}{c} 1\times 1,64\\ 3\times 3,64\\ 1\times 1,256 \end{array}\right]$ × 3	$\left[\begin{array}{c} 1\times 1,64\\ 3\times 3,64\\ 1\times 1,256 \end{array}\right]$ × 3	$\left[\begin{array}{c} 1\times 1,64\\ 3\times 3,64\\ 1\times 1,256 \end{array}\right]$ × 3	$\left[\begin{array}{c} 1\times 1,64\\ 3\times 3,64\\ 1\times 1,256 \end{array}\right]$ × 3
Stage 2	$\left[\begin{array}{c} 1\times 1,128\\ 3\times 3,128\\ 1\times 1,512 \end{array}\right]$ × 4	$\left[\begin{array}{c} 1\times 1,128\\ 3\times 3,128\\ 1\times 1,512 \end{array}\right]$ × 4	$\left[\begin{array}{c} 1\times 1,128\\ 3\times 3,128\\ 1\times 1,512 \end{array}\right]$ × 4	$\left[\begin{array}{c} 1\times 1,128\\ 3\times 3,128\\ 1\times 1,512 \end{array}\right]$ ×4	$\left[\begin{array}{c} 1\times 1,128\\ 3\times 3,P\\ 1\times 1,512 \end{array}\right]$ × 4	$\left[\begin{array}{c} 1\times 1,P\\ 3\times 3,P\\ 1\times 1,512 \end{array}\right]$ × 4	$\left[\begin{array}{c} 1\times 1,P\\ 3\times 3,P\\ 1\times 1,512 \end{array}\right]$ × 4
Stage 3	$\left[\begin{array}{c} 1\times 1,P\\ 3\times 3,P\\ 1\times 1,1024 \end{array}\right]$ × 6	$\left[\begin{array}{c} 1\times 1,P\\ 3\times 3,P\\ 1\times 1,1024 \end{array}\right]$ × 6	$\left[\begin{array}{c} 1\times 1,P\\ 3\times 3,P\\ 1\times 1,1024 \end{array}\right]$ × 6	$\left[\begin{array}{c} 1\times 1,P\\ 3\times 3,P\\ 1\times 1,1024 \end{array}\right]$ × 6	$\left[\begin{array}{c} 1\times 1,P\\ 3\times 3,P\\ 1\times 1,1024 \end{array}\right]$ × 6	$\left[\begin{array}{c} 1\times 1,P\\ 3\times 3,P\\ 1\times 1,1024 \end{array}\right]$ × 6	$\left[\begin{array}{c} 1\times 1,P\\ 3\times 3,P\\ 1\times 1,1024 \end{array}\right]$ × 6
Stage 4	$\left[\begin{array}{c} 1\times 1,P\\ 3\times 3,P\\ 1\times 1,2048 \end{array}\right]$ × 3	$\left[\begin{array}{c} 1\times 1,P\\ 3\times 3,P\\ 1\times 1,2048 \end{array}\right]$ × 3	$\left[\begin{array}{c} 1\times 1,P\\ 3\times 3,P\\ 1\times 1,2048 \end{array}\right]$ × 3	$\left[\begin{array}{c} 1\times 1,P\\ 3\times 3,P\\ 1\times 1,2048 \end{array}\right]$ × 3	$\left[\begin{array}{c} 1\times 1,P\\ 3\times 3,P\\ 1\times 1,2048 \end{array}\right]$ × 3	$\left[\begin{array}{c} 1\times 1,P\\ 3\times 3,P\\ 1\times 1,2048 \end{array}\right]$ × 3	$\left[\begin{array}{c} 1\times 1,P\\ 3\times 3,P\\ 1\times 1,2048 \end{array}\right]$ × 3

*In each reduction level, P indicates the block position where channels were pruned*.

Table 3 shows the computational requirements and classification performances of the models generated from the simplified networks. NAR with ϕ = 2 generated a network with 70% reduction in computational requirements compared to the original network. Additionally, the AUC value obtained by the simplified model is the same as that achieved by the original model. ResRep also was able to generate simplified networks with no loss of AUC performance. However, as is shown in the table, these networks had higher computational requirements than the networks generated by NAR. Further, the IR strategy achieved competitive results as compared to ResRep, although it is a relatively simple approach. NAR has attained an overall better performance (smaller G) than IR for the same AUC. Further, it is noticeable that when the input image is reduced bellow a certain size (e.g., 119×119), the AUC of IR is significantly impacted.

**Table 3:** AUC, Giga- (G) correspondent to model input size, number of parameter layers, and total of model layers of ResNet50 V2 and simplified networks by ResRep, IR, and NAR.

**CNN**	**AUC**	**G**	**Input size**	**Param. layers**	**# of layers**
ResNet50 V2 (53)	0.86	9.65	240 ×240	50	225
ResNet ResRep ϵ = 0.82	0.87	8.34	240 ×240	50	225
ResNet ResRep ϵ = 0.84	0.82	7.91			
ResNet ResRep ϵ = 0.86	0.84	7.63			
ResNet ResRep ϵ = 0.88	0.81	7.68			
ResNet ResRep ϵ = 0.90	**0.86**	**7.27**			
ResNet ResRep ϵ = 0.92	0.69	6.10			
ResNet ResRep ϵ = 0.94	0.73	6.09			
ResNet50 V2 IR 1	0.88	7.90	209 ×209	50	225
ResNet50 V2 IR 2	0.88	5.83	181 ×181		
ResNet50 V2 IR 3	**0.86**	**4.24**	157 ×157		
ResNet50 V2 IR 4	0.84	3.49	137 ×137		
ResNet50 V2 IR 5	0.81	2.56	119 ×119		
ResNet50 V2 IR 6	0.79	2.03	104 ×104		
ResNet NAR ϕ = 1	0.84	5.15	209 ×209	42	170
ResNet NAR ϕ = 2	**0.86**	**2.96**	181 ×181	36	160
ResNet NAR ϕ = 3	0.80	1.53	157 ×157	30	134

*Bold values are those with good quality/performance trade offs*.

An interesting configuration of ResRep occurred when ϵ was set to 0.90. The computational requirements of the simplified network was 75.0% of that of the original network, and the simplified network attained an equivalent AUC level. However, when a higher simplification value was used, there was a significant drop in AUC. For the same AUC values (e.g., 0.86), NAR generated CNNs with smaller computational requirements.

### 3.2. NAR and IR Performance for the Inception V4 CNN

This set of experiments measures the performance of NAR and IR with Inception V4 (54). The Inception is a deeper network than ResNet50 V2 and has a higher computational cost, thus it is another interesting case for evaluating our approach. We unfortunately have not been able to use ResRep to simplify the Inception. This CNN has a more complex architecture with multiple shortcuts and the ResRep code/documentation available does not implement Inception neither it provides clear directions on how to apply the method to other complex architectures (27).

The results of the NAR simplified networks as the ϕ parameter is varied are shown in Table 4. First, it is noticeable that the original Inception showed a better classification performance as compared to ResNet (0.92 vs. 0.87). As compared to the IR strategy, NAR has again attained better performance for the same AUC level. Once again, for the best AUC score of each strategy and 0.87 AUC values, NAR requires, respectively, about 2.35 × and 4.93 × less FLOPs to compute an inference. These observations once again show the importance of a balanced compound network reduction as performed by NAR.

**Table 4:** AUC, Giga-FLOPs (GFLOPs) correspondent to input sizes, number of parameter layers, and total layers of Inception V4 and simplified networks produced by NAR.

**CNN**	**AUC**	**G**	**Input size**	**Param. layers**	**# of layers**
Inception V4 (54)	0.92	15.48	240 ×240	245	861
Inception IR 1	**0.91**	**9.80**	209 ×209	245	861
Inception IR 2	0.89	6.64	181 ×181		
Inception IR 3	0.88	4.79	158 ×158		
Inception IR 4	0.87	2.76	137 ×137		
Inception IR 5	0.86	1.92	119 ×119		
Inception IR 6	0.77	1.14	104 ×104		
Inception NAR ϕ = 1	**0.91**	**8.14**	209 ×209	206	723
Inception NAR ϕ = 2	**0.92**	**4.17**	181 ×181	179	627
Inception NAR ϕ = 3	0.90	2.21	158 ×158	145	507
Inception NAR ϕ = 4	0.88	1.02	137 ×137	123	429
Inception NAR ϕ = 5	0.87	0.56	119 ×119	101	351
Inception NAR ϕ = 6	0.84	0.28	104 ×104	91	315

*Bold values are those with good quality/performance trade offs*.

Further, the NAR simplified version had a far better trade-off in terms of the GFLOPs required to attain a certain AUC when compared to ResNet. For instance, NAR ϕ = 5 reached an AUC of 0.87 with only 0.56 GFLOPs. A comparable performance level required at least 8.34 GFLOPs and 2.96 GFLOPs with the simplified ResNet networks, respectively, with ResRep and NAR. The results also show that the simplified Inception V4 can sustain the same AUC level as the original network with a computational cost reduction of about 4 × (NAR ϕ = 2).

## 4. Discussion

Overall, the experimental evaluation shows that it is possible to simplify a classification CNN to reduce its computational requirements in the inference phase, while maintaining model performance comparable to the original CNN. The ResNet50 models generated by ResRep with ϵ = 0.90 and by NAR with ϕ = 2 practically achieved the same AUC scores as the models from the original ResNet50 V2 network and were computationally 1.32 × and 3.26 × cheaper, respectively. Our method, NAR, produced more efficient networks than ResRep. We attribute this improvement to the fact that NAR employs an approach that simplifies the multiple components of a network in a more balanced manner. The analysis of the shape structure of the simplified CNNs with both methods (shown in Tables 1, 2) highlights the main differences among their simplification strategies. ResRep mainly modified the latest layers of the CNNs, while NAR carried out a more homogeneous simplification over all of the network stages. Previous work (28, 50, 51) demonstrated that such a balance among the CNN components is important to maximize classification quality. The better compromises of NAR vs. IR strategy also demonstrate in practice that the compound reduction performed by the first is important into maximizing AUC while reducing the FLOPs demand.

This observation aligns well with the goal of our NAR method, which is to modify the width, depth, and input resolution components of a network together and in a simple way. Additionally, NAR is easier to use, requiring few alterations to an original network, without the need to change the training dynamics. ResRep, on the other hand, is harder to use as it requires changing the network with extra layers and also includes new CNN training penalties etc. This is even harder with deeper CNNs that are becoming more popular.

In the experiments with Inception V4, which is a deeper network than ResNet50, we observed that the original Inception V4 has achieved overall better AUC than the original ResNet50, but it was about 1.6 × more expensive. The simplified version generated by NAR with ϕ = 5 achieved an AUC value of 0.87, which is comparable to the original ResNet50 network, and was faster than the simplified ResNet with the same AUC value; the simplified Inception V4 model required 0.56 GFLOPs while the simplified ResNet50 model required 2.96 GFLOPs (about 5.3 × more expensive). Our experimental evaluation suggests that during the development of a deep learning network, it may be better to focus on the classification performance of the network and worry less about its computational requirements and further apply a network simplification step after the network architecture has been fine-tuned for classification performance.

In our work we used classification of TILs in whole slide images as the driving application use case. We expect that our method can be generalized to other classification problems in digital pathology. Characterization of TIL patterns in whole slide images is an important use case. Multiple studies have shown that there is a correlation between the density and spatial organization of TILs and clinical outcomes (37, 38, 56, 57). Characterizations of TIL patterns can lead to better understanding of cancer mechanisms and improve cancer staging (58). There is an increasing number of computational pathology approaches to generate such characterizations (34, 59, 60).

Applications of deep learning methods for TIL analysis on a large number of whole slide images is desirable, as they can result in a better understanding of TIL patterns. It is important to employ effective and efficient deep learning methods in order to facilitate such applications. We have shown that our approach can reduce computational requirements by roughly of 4 × without impacting overall classification quality for two real-world CNN networks. This is a significant improvement in execution cost and can enable a broader use of these techniques in digital pathology. We also believe this paper opens multiple interesting directions for future work. First, as briefly discussed, it would be important to evaluate a larger number of CNN architectures to analyze how simplification methods would affect their AUC and count. This could answer the question regarding whether the developer should worry or not about the FLOPs required or network complexity during the development, or if this could be resolved by simplification methods in all cases. Second, we also want to expand this analysis with additional pathology image analysis applications, including not only additional classification applications but also segmentation tasks, for instance.

## Data Availability Statement

The raw data supporting the conclusions of this article will be made available by the authors, without undue reservation.

## Author Contributions

AM implemented the code, performed the experiments, and organized the dataset. AM, TK, and GT performed the experimental analysis. AM and GT wrote the first draft of the manuscript. AM, TK, JK, RF, JS, and GT wrote the final manuscript. All authors contributed to conception and design of the study. All authors contributed to manuscript revision, read, and approved the submitted version.

## Funding

This work was supported in part by 1UG3CA225021 from the NCI, R01LM011119-01 and R01LM009239 from the NLM, CNPq, Capes/Brazil Grants PROCAD-183794, FAPEMIG, PROCAD/UFMG, K25CA181503, and U01CA242936 from National Institute of Health and generous donations from Bob Beals and Betsy Barton. This work used the Extreme Science and Engineering Discovery Environment (XSEDE), which is supported by National Science Foundation Grant Number ACI-1548562. Specifically, it used the Bridges system, which is supported by NSF award number ACI-1445606, at the Pittsburgh Supercomputing Center (PSC).

## Conflict of Interest

The authors declare that the research was conducted in the absence of any commercial or financial relationships that could be construed as a potential conflict of interest.

## Publisher's Note

All claims expressed in this article are solely those of the authors and do not necessarily represent those of their affiliated organizations, or those of the publisher, the editors and the reviewers. Any product that may be evaluated in this article, or claim that may be made by its manufacturer, is not guaranteed or endorsed by the publisher.
