# Supplementary material for: Building Efficient CNN Architectures for Histopathology Images Analysis: A Case-Study in Tumor-Infiltrating Lymphocytes Classification
Source: Front Med (Lausanne). 2022 May 31;9:894430. doi: 10.3389/fmed.2022.894430 (PMC9197439; doi:10.3389/fmed.2022.894430)
Supplement: Supplementary file 1 [file Data_Sheet_1.PDF]

frontiers

Supplementary Material

1 WHOLE SLIDE IMAGE DATA

All tumor WSI images used in our evaluation were retrieved from The Cancer Genome Atlas (TCGA). The number of slides and types of tissues used in our experiments are given by Tables S1 and S2.

Acronym	Tumor type
BLCA	Bladder urothelial carcinoma
BRCA	Breast invasive carcinoma
CESC	Cervical squamous cell carcinoma and endocervical adenocarcinoma
COAD	Colon adenocarcinoma
PAAD	Pancreatic adenocarcinoma
PRAD	Prostate adenocarcinoma
READ	Rectum adenocarcinoma
SKCM	Skin Cutaneous Melanoma
STAD	Stomach adenocarcinoma
UCEC	Uterine Corpus Endometrial Carcinoma

Table S1. Tumor types.

	prad	ucec	blca	stad	skcm	coad	brca	cesc	paad	read
Train/Val	4	13	4	9	8	4	7	5	1	1
Test	0	0	0	0	2	0	2	1	0	0
Total	4	13	4	9	10	4	9	6	1	1

Table S2. Tissue types and number of slides for each.

WSI ID	Total patches	Positive patches	% Positive	Magnification
TCGA-AM-5820-01Z-00-DX1	4350	139	3.20	40x
TCGA-AP-A0LH-01Z-00-DX1	17142	6770	39.49	20x
TCGA-AP-A1DM-01Z-00-DX1	8976	3035	33.81	20x
TCGA-AU-6004-01Z-00-DX1	3142	1545	49.17	40x
TCGA-AY-6196-01Z-00-DX1	11054	763	6.90	40x
TCGA-BG-A0M4-01Z-00-DX1	16332	1573	9.63	40x
TCGA-BG-A0MQ-01Z-00-DX1	12157	2627	21.61	40x
TCGA-BG-A0W1-01Z-00-DX1	14540	3580	24.62	40x
TCGA-BH-A0BR-01Z-00-DX1	5429	71	1.31	40x
TCGA-BH-A202-01Z-00-DX1	11618	736	6.33	40x
TCGA-BL-A13J-01Z-00-DX1	2154	108	5.01	40x
TCGA-BR-6709-01Z-00-DX1	9792	6275	64.08	40x
TCGA-BR-8058-01Z-00-DX1	10295	1719	16.70	40x
TCGA-BR-8682-01Z-00-DX1	10355	2066	19.95	40x
TCGA-BS-A0UF-01Z-00-DX1	11965	2317	19.36	40x
TCGA-C4-A0F6-01Z-00-DX1	1056	108	10.23	40x
TCGA-C5-A1MH-01Z-00-DX1	4963	1841	37.09	40x
TCGA-C5-A7CK-01Z-00-DX1	4396	516	11.74	40x
TCGA-D1-A16N-01Z-00-DX1	9840	1181	12.00	40x
TCGA-D1-A176-01Z-00-DX1	14050	3406	24.24	40x
TCGA-D3-A8GJ-06Z-00-DX1	13616	5026	36.91	40x
TCGA-D5-6534-01Z-00-DX1	9607	3095	32.22	40x
TCGA-D7-6519-01Z-00-DX1	11724	2314	19.74	40x
TCGA-D8-A1XD-01Z-00-DX1	10360	359	3.47	40x
TCGA-D8-A1XU-01Z-00-DX1	6666	191	2.87	40x
TCGA-DS-A7WF-01Z-00-DX1	17504	1266	7.23	40x
TCGA-E9-A3X8-01Z-00-DX1	3283	116	3.53	40x
TCGA-EE-A2GS-01Z-00-DX1	5112	128	2.50	40x
TCGA-EE-A2MH-01Z-00-DX1	1889	879	46.53	40x
TCGA-EJ-5497-01Z-00-DX1	11290	458	4.06	40x
TCGA-EJ-5530-01Z-00-DX1	6617	225	3.40	40x
TCGA-EO-A22S-01Z-00-DX1	5786	1037	17.92	40x
TCGA-EW-A1J1-01Z-00-DX1	11514	2039	17.71	40x
TCGA-EW-A1PF-01Z-00-DX1	6943	584	8.41	40x
TCGA-EY-A2OO-01Z-00-DX1	6552	100	1.53	40x
TCGA-EY-A3L3-01Z-00-DX1	7863	288	3.66	40x
TCGA-EY-A549-01Z-00-DX1	5332	269	5.05	40x
TCGA-F5-6864-01Z-00-DX1	4489	1662	37.02	40x
TCGA-FI-A2EY-01Z-00-DX1	11258	342	3.04	40x
TCGA-FR-A728-01Z-00-DX1	3759	165	4.39	40x
TCGA-FR-A7U8-01Z-00-DX1	8877	997	11.23	40x
TCGA-HC-8264-01Z-00-DX1	1365	37	2.71	40x
TCGA-HG-A9SC-01Z-00-DX1	11194	2915	26.04	40x
TCGA-HU-8610-01Z-00-DX1	1117	179	16.03	20x
TCGA-HU-A4GP-01Z-00-DX1	1914	969	50.63	20x
TCGA-OD-A75X-06Z-00-DX1	11220	1013	9.03	40x
TCGA-S5-AA26-01Z-00-DX1	5393	827	15.33	40x
TCGA-US-A77G-01Z-00-DX1	13611	1784	13.11	40x
TCGA-VQ-A8DT-01Z-00-DX1	11344	3979	35.08	40x
TCGA-VQ-A8P8-01Z-00-DX1	10145	2397	23.63	40x
TCGA-VQ-A94U-01Z-00-DX1	11672	1115	9.55	40x
TCGA-VS-A8EJ-01Z-00-DX1	886	44	4.97	40x
TCGA-W3-AA1V-01Z-00-DX1	9193	535	5.82	40x
TCGA-XF-AAMX-01Z-00-DX1	11489	1816	15.81	40x
TCGA-XV-AAZW-01Z-00-DX1	20128	805	4.00	40x
TCGA-YL-A8HL-01Z-00-DX1	14567	531	3.65	40x

Table S3. WSIs used for training.

WSI ID	Total patches	Positive patches	% Positive	Magnification
TCGA-D9-A149-01Z-00-DX1	8618	1691	19.62	40x
TCGA-AO-A0JI-01Z-00-DX1	4708	589	12.51	20x
TCGA-C8-A12K-01Z-00-DX1	7217	12	0.17	40x
TCGA-EE-A3AF-01Z-00-DX1	10778	331	3.07	40x
TCGA-MY-A913-01Z-00-DX1	4885	939	19.22	40x

Table S4. WSIs used for testing.
